# Surgery in Nonalcoholic Cirrhosis: Clinical Outcomes, Healthcare Utilization, and Cost Analysis

**DOI:** 10.7759/cureus.39762

**Published:** 2023-05-31

**Authors:** Christopher Tait, Ankoor H Patel, You Li, Carlos D Minacapelli, Vinod Rustgi

**Affiliations:** 1 Division of Gastroenterology and Hepatology, Department of Medicine, Rutgers Robert Wood Johnson Medical School, New Brunswick, USA; 2 Department of Medicine, Rutgers Robert Wood Johnson Medical School, New Brunswick, USA

**Keywords:** septic shock [ss], surgical risk factors, cost effectiveness analysis, surgery outcomes, cirrohsis

## Abstract

Background: Patients with cirrhosis are at increased risk of complications following surgery due to multiple factors, including portal hypertension and alterations in hemostasis. Improvements in perioperative management as well as risk stratification scores have helped improve outcomes, but gaps remain in our understanding of the cost and morbidity of cirrhotic patients who undergo surgery.

Methods: We conducted a case-control study using the IBM Electronic Health Record (EHR) MarketScan Commercial Claims (MSCC) database from January 1, 2007 to December 31, 2017. Nonalcoholic cirrhotic patients who underwent surgery were identified based on International Classification of Diseases, Ninth Revision (ICD-9)/Tenth Revision (ICD-10) codes for multiple surgical categories and matched with controls with cirrhosis who did not undergo surgery in this time period. A total of 115,512 patients were identified with cirrhosis, of whom 19,542 (16.92%) had surgery. Medical history and comorbidities were compiled, and outcomes in the six-month period following surgery were analyzed between matched groups. A cost analysis was performed based on claims data.

Results: Nonalcoholic cirrhotic patients who underwent surgery had a higher comorbidity index at baseline compared with controls (1.34 vs. 0.88, P<0.0001). Mortality was increased in the surgery group (4.68% vs. 2.38%, P<0.001) in the follow-up period. The surgical cohort had higher rates of adverse hepatic outcomes, including hepatic encephalopathy (5.00% vs. 2.50%, P<0.0001), spontaneous bacterial peritonitis (0.64% vs. 0.25%, P<0.001), and higher rates of septic shock (0.66% vs. 0.14%, P<0.001), intracerebral hemorrhage (0.49% vs. 0.04%, P<0.001), and acute hypoxemic respiratory failure (7.02% vs. 2.31%, P<0.001). Healthcare utilization analysis revealed increased total claims per patient in the surgical cohort (38.11 vs. 28.64, P<0.0001), higher inpatient admissions (6.05 vs. 2.35, P<0.0001), more outpatient visits (19.72 vs. 15.23, P<0.0001), and prescription claims per patient (11.76 vs. 10.61, P<0.0001) in the postsurgical period. The likelihood of at least one inpatient stay was higher in the surgical cohort (51.63% vs. 22.32%, P<0.0001), and inpatient stays were longer (4.99 days vs. 2.09 days, P<0.0001). The total cost of health services was significantly increased per patient in the postoperative period for patients undergoing surgery ($58,246 vs. $26,842, P<0.0001), largely due to increased inpatient costs ($34,446 vs. $10,789, P<0.0001).

Conclusion: Nonalcoholic cirrhotics undergoing surgery experienced worse outcomes with respect to adverse hepatic events and complications, including septic shock and intracerebral hemorrhage. Claims and cost analysis showed a significant increase in health expenditure in the surgical group, largely due to the cost of more frequent and longer inpatient admissions.

## Introduction

Cirrhosis is an end-stage pathologic process resulting from multiple etiologies, including chronic viral hepatitis, alcoholic liver disease, and metabolic liver diseases such as nonalcoholic steatohepatitis (NASH). All of these conditions lead to chronic injury through progressive inflammation and subsequent fibrosis, with the development of portal hypertension and progressive chronic liver failure [1]. The main source of morbidity results from portal hypertension and its associated sequelae, but cirrhotic patients exhibit other complications as a result of chronic liver dysfunction, including alterations in hemostasis with an increased risk of both bleeding and thrombosis [2]. The global prevalence of cirrhosis has increased over the last 30 years, driven by rising cases of NASH, alcoholic liver disease, and viral hepatitis [3]. Despite this increased prevalence, cirrhosis-related mortality rates have significantly decreased due to advances in treatment modalities and medical care [1].

Patients with cirrhosis are surviving longer with more advanced disease and are more frequently developing comorbidities that require emergent or elective surgery [1]. The cirrhotic patient population undergoes many of the same types of elective or emergent surgeries as the general population, as well as additional population-specific surgeries, including hepatectomy or liver transplantation for the management of hepatocellular carcinoma, for which they are at significantly increased lifetime risk [4]. Surgical outcomes in cirrhotics have traditionally been associated with high morbidity and mortality, with earlier series citing 30-day mortality rates as high as 67% for intra-abdominal surgeries on patients with advanced disease [5]. Improvements in patient selection and perioperative management have led to improved surgical survival; however, perioperative mortality remains two to 10 times higher in cirrhotics compared with healthy controls as correlated with severity of liver dysfunction [6]. Risk prediction algorithms have been devised to help perform procedural risk. These algorithms factor in additional risk features, including the type of surgical procedure and the type of anesthesia used [7]. Despite risk assessment tools and therapeutic improvements, perioperative evaluation and management have not been standardized, and there is little prospective data available to inform clinical decision-making.

 Unique pathophysiologic changes seen in cirrhosis contribute to worse outcomes compared with healthy patients. Protein synthetic dysfunction due to liver disease results in significant sarcopenia and malnutrition, which hinder wound healing and physical recovery from surgery [1]. Portal hypertension, which progresses with more advanced disease, raises the risk of bleeding from collateral circulation, contributes to thrombocytopenia through splenic sequestration, and influences thrombopoietin signaling [2]. Other disruptions in the coagulation cascade increase the likelihood of both perioperative bleeding and clotting. The severity of liver disease is the most important factor in determining perioperative mortality risk and is commonly assessed by the Child-Turcotte-Pugh (CTP) class score and the Model for End-Stage Liver Disease (MELD) score. A summary of the factors used in calculating these scores, as well as factors used in calculating the most common surgical risk scores used in cirrhosis (American Society of Anesthesiology (ASA) classification, Mayo Risk Calculator, and Veterans Outcomes and Costs Associated with Liver Disease Penn (VOCAL-Penn) scoring system), is summarized in Table 1.

In addition to the severity of liver disease, other significant risk factors identified include patient characteristics, the type of surgery, and the type of anesthesia used. Pertinent patient-specific risk factors noted have included patient age and ASA class as independent risk factors [8]. ASA class describes patients on a class of 1-6 in escalating severity of systemic disease. The Mayo risk score incorporates the MELD score, ASA class, and patient age to estimate mortality up to 1 year. The Mayo score at 30 and 90 days correlates linearly with MELD scoring, with modest improvement in predictive accuracy [8]. Some limitations apply to the Mayo score, which was validated in patients who underwent major digestive, orthopedic, or cardiovascular surgery and was not derived to predict outcomes in other elective procedures. It is also likely that improvements in surgical outcomes since 2007, which is when the Mayo score was derived, may weaken its predictive accuracy. A more recently derived predictive tool, the VOCAL-Penn score, was derived from a large cohort of 3,785 cirrhotic patients with primarily CP Class A cirrhosis and MELD <9 who underwent a broad range of surgical procedures [7]. This score combines several features of Child-Pugh scoring with patient age, ASA score, the type of surgery involved, and body mass index >30, which has been found to be a protective factor against postoperative mortality [9]. The VOCAL-Penn model showed superior performance to MELD, CTP, and Mayo scores at 30, 90, and 180-day postoperative mortality predictions but perhaps has limitations about generalized applicability due to the subject population studied [7].

Significant gaps remain in our understanding of the postoperative costs and morbidity associated with surgical procedures in cirrhotic patients. Few of the published series involved large cohorts, analyzed cirrhotics on the type or frequency of surgery in these cohorts, or provided insight into financial cost as an outcome [6]. Identifying risk features for poor outcomes and cost burdens in the larger population of evolving cirrhotic patients is a crucial goal in informing future surgical risk and health burden.

The aim of this retrospective study is to analyze these features at scale utilizing the IBM® Electronic Health Record (EHR) MarketScan database. This administrative claims database provides de-identified claims and utilization records for patients with employer-based health insurance and select Medicare and Medicaid patients. While not entirely representative nationally, selection from this database allows for the analysis of a very large cohort of patients and can provide outcomes data on a much larger selected proportion of the population than published in previous series. Our goal is to identify baseline risk features in cirrhotic patients who are planning to undergo surgery, analyze and compare outcomes in a six-month follow-up period, and determine health costs based on health claims data in this time period. A subgroup analysis by type of surgery is presented to help differentiate risk by surgery type.

## Materials and methods

Data source

We conducted a case-control study using the IBM® EHR MarketScan® Commercial Claims (MSCC) database from January 1, 2007 to December 31, 2017. Data included in the MSCC database characterized national healthcare records from government and public organizations, large employers, and health plans annually. The MSCC database included de-identified longitudinal individual-level claims data across inpatient, outpatient, and prescription drug services from approximately 350 payers annually. The MSCC database provided the total gross cost of care, which represents the amounts eligible for payment after applying pricing guidelines, such as discounts and fee schedules, and before applying coordination of deductibles, benefits, and copayments. Records from MSCC were de-identified data and compliant with all United States patient confidentiality requirements, including the Health Insurance Portability and Accountability Act of 1996. The protocol of this study was approved by the Internal Review Board (IRB) of Rutgers Robert Wood Johnson Medical School.

Study sample

The study sample was created using inpatient admissions and outpatient records from January 1, 2007 to December 31, 2017, including adult (18+) patients with nonalcoholic cirrhosis. Nonalcoholic cirrhosis was defined as one inpatient admission or outpatient service diagnosis International Classification of Diseases, Ninth Revision (ICD-9) code 571.5 or Tenth Revision (ICD-10) codes K74.6, K74.60, and K74.69. We then searched by surgery status among all MarketScan® participants as at least one primary or secondary ICD-9/ICD-10 code for abdominal/gastrointestinal, genitourinary/ gynecologic, cardiac, thoracic, head and neck, or orthopedic surgeries. Using the surgery and cirrhosis cohorts, we identified three comparator groups: (1) patients with both cirrhosis and surgery, (2) patients with cirrhosis without surgery, and (3) patients without cirrhosis with surgery.

The case cohort included patients with both cirrhosis and surgery diagnosis, whereas the control cohort included patients with only cirrhosis (i.e., no surgery cohort). The index date was defined as the earliest date of (1) cirrhosis diagnosis (for patients with cirrhosis only) and (2) surgery date for patients with cirrhosis who underwent surgery. For patients with cirrhosis who underwent surgery, only patients with surgery no earlier than first cirrhosis index (i.e., surgery performed after cirrhosis was identified) were included. Each participant’s baseline period was defined as six months prior to the selected index date, while the follow-up period was defined as six months following the defined index date. A subgroup analysis was performed on the surgery in cirrhosis patients based on type of surgery based on ICD-9 and ICD-10 codes for nine major surgical subtypes: cholecystectomy, appendectomy, gastrectomy, colectomy, hernia repair, mastectomy, total knee replacement, total hip replacement, and aortic valve replacement.

Study variables

Index date records were utilized to obtain demographics, including age, gender, region of residence, and healthcare insurance plan. Using ICD-9/ICD-10 codes acquired from inpatient admissions and outpatient services, a medical history comorbidity profile was calculated for each participant during the baseline period. The comorbidity profile included ascites, hepatic encephalopathy (HE), spontaneous bacterial peritonitis (SBP), shock, acute hypoxemic respiratory failure (AHRF), acute kidney injury (AKI), hepatorenal syndrome (HRS), pneumonia, aspiration, intracerebral bleeding, intracerebral thrombosis, pulmonary embolism (PE), ventilator dependence, septic shock, sepsis, and acute tubular necrosis (ATN). In addition, the total weighted Charlson Comorbidity Index (CCI) score was calculated for each participant using the baseline period records [10]. Similarly, an outcome profile was measured for the six months following the index date for those same baseline variables. In addition, the follow-up profile included blood product transfusion, packs of blood product transfusion, platelets, average platelets used, albumin, and average albumin used. For variables measured both before and after surgery, postoperative variables were captured only if the diagnosis was new in the postoperative period. 

Matching procedure

Propensity score matching was used to account for the comparability between cases and controls on baseline demographics and comorbidity profiles. A propensity score was estimated for each participant using a multivariate logistic regression model, with cirrhosis with/without surgery and surgery in patients without cirrhosis status as the outcome and age group, gender, region of residence, type of health insurance, smoke, alcohol, and length of enrollment post-index as covariates (Table 1). Cases were matched 1:1 to the nearest neighbor estimated propensity score without replacement strategy (i.e., GREEDY algorithm) while matching exactly on age group and gender [11].

**Table 1:** Predictive models of surgical outcomes in cirrhosis. CTP: Child-Turcotte-Pugh; ASA: American Society of Anesthesiology; MELD: Model for End-Stage Liver Disease; VOCAL-Penn: Veterans Outcomes and Costs Associated with Liver Disease; NAFLD: nonalcoholic fatty liver disease.

Predictive model	Formula/description
CTP score	Encephalopathy grade: None (1), stage 1-2 (2), stage 3-4 (3); Ascites level: absent (1), slight (2), moderate or severe (3); Serum albumin (g/dL): >3.5 (1), 2.8-3.5 (2), <2.3 (3); Total bilirubin (mg/dL): <2 (1), 2-3 (2), >3 (3)
ASA classification	Class 1: Normal healthy patient; Class 2: mild systemic disease; Class 3: severe systemic disease that is not life-threatening; Class 4: severe systemic disease that is a constant threat to life; Class 6: moribund and not expected to survive without the operation; Class 6: brain-dead patient/organ donor
MELD score	Score weighted for: Serum bilirubin (mg/dL), serum INR, serum creatinine (mg/dL)
Mayo risk score	Age classification of cirrhosis (alcoholic, cholestatic, viral, other), MELD score, ASA classification
VOCAL-Penn score	Age serum albumin (g/dL), total bilirubin (mg/dL), platelet count (x1000/mL), BMI >30, presence of NAFLD, ASA score, emergency surgery qualification, surgery type (laparoscopic abdominal, open abdominal, abdominal wall, vascular, major orthopedic, chest/cardiac)

Healthcare utilization and costs

Healthcare utilization was measured by the number of claims per patient for emergency department (ED) visits, inpatient admissions, pharmaceutical prescriptions, and outpatient visits. The healthcare burden was defined by the total annual service-specific healthcare costs generated from claims related to ED visits, inpatient admissions, pharmaceutical prescriptions, and outpatient visits. To measure the burden associated with either cirrhosis with/without surgery or surgery without cirrhosis, we totaled the healthcare utilization and cost parameters over the six months post-index. Utilization and cost parameters over the six months following the index date for cirrhosis with/without surgery and surgery without cirrhosis were quantified.

Healthcare utilization was measured using the mean, median, and 25th/75th percentiles of the number of claims per patient for ED visits, inpatient admissions, pharmaceutical prescriptions, and outpatient visits. In addition, the prevalence of having at least one ED visit, inpatient admission, and outpatient visit was estimated. Of note, inpatient admission data included if the patient was hospitalized at any point after the index surgery, including immediately postsurgery. The average length of inpatient stay per patient for all participants was also measured for the six months following the index date. The mean, median, and 25th/75th percentiles of the healthcare costs were calculated for both the overall and service-specific costs for the six months following the index date. A subanalysis was performed and quantified age-specific healthcare cost comparisons between cirrhosis with/without surgery and surgery without cirrhosis for all expenditure variables post six months following the index date. All cost estimates were adjusted to the 2019 United States dollar (US$) using the medical care commodities component of the Consumer Price Index.

Statistical analysis

Baseline characteristics and comorbidity profiles were compared, before and after matching, for those with cirrhosis with/without surgery and surgery without cirrhosis, using the standardized differences of means and proportions. We indicated small, medium, and large differences between means and proportions of the compared groups by using standardized difference cutoffs of 0.2, 0.5, and 0.8, respectively (Table 1) [12,13]. Wald chi-square tests were used to test the associations between cirrhosis with/without surgery and surgery without cirrhosis status, patients’ categorical characteristics, and comorbidity profiles in the unmatched sample. Wilcoxon signed-rank tests were utilized to compare all continuous healthcare costs and utilization. The McNemar test was used to compare dichotomous parameters of healthcare utilization in the six months following the index date. P-values were obtained from Wilcoxon ranked test for continuous variables and McNemar test for binary variables. P<0.05 was considered statistically significant. The study analysis was performed using SAS 9.4 software (SAS Institute, Cary, NC, United States).

## Results

Perioperative and baseline characteristics

**Figure 1:**
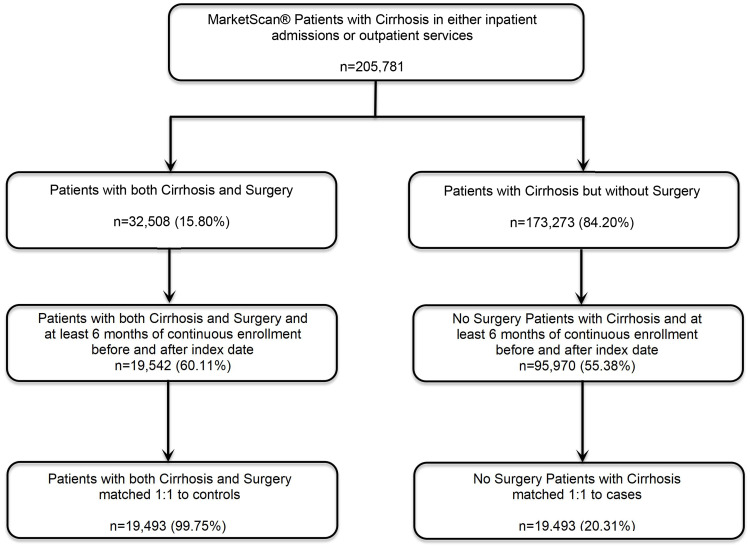
Diagram for study sample selection.

The study sample included 115,512 participants with cirrhosis in the MSCC database who met the inclusion criteria of continuous enrollment for six months before and after the index date (Figure 1). Of the 115,512 patients with a cirrhosis diagnosis, 16.92% (19,542) had surgery. Of the 19,542 surgery cases identified from 2007 to 2017, 19,493 were matched 1:1 using propensity scores to the control group with cirrhosis alone.

Between-group differences in the unmatched and matched samples are summarized in Table 2. Patients in the surgery cohort had higher comorbidities compared to the no surgery cohort (CCI 4+, surgery vs. no surgery, 7.77% vs. 3.25%; P<0.0001). The cirrhotics who then underwent surgery had a higher prevalence of ascites (24.19% vs. 17.51%), HE (11.59% vs. 7.31%), SBP (2.02% vs. 1.09%), HRS (1.48% vs. 0.80), esophageal varices (23.60% vs. 20.19), and portal hypertension (20.38% vs. 16.46%) at baseline prior to surgery compared to cirrhotics who did not undergo surgery, all P-values <0.001. The distribution of all baseline characteristics was balanced between the matched groups (Table 2).

**Table 2:** Baseline characteristics of unmatched and matched cirrhosis preoperative groups. HE: hepatic encephalopathy; AHRF: acute hypoxemic respiratory failure; AKI: acute kidney injury; HRS: hepatorenal syndrome; PE: pulmonary embolism; ATN: acute tubular necrosis; SBP: spontaneous bacterial peritonitis.

Demographic features	Unmatched			Matched		
	Cirrhosis with surgery	Cirrhosis without surgery	P-value	Cirrhosis with surgery	Cirrhosis without surgery	P-value
	(n=19,542)	(n=95,970)		(n=19,493)	(n=19,493)	
Age (years), mean (SD)	53.63 (9.06)	52.56 (10.36)	<0.0001	53.65 (9.05)	53.79 (9.29)	0.1360
Age group (years), n (%)			<0.0001			1.0000
<18	139 (0.71)	1,220 (1.27)		139 (0.71)	139 (0.71)	
18-34	740 (3.79)	5,112 (5.33)		736 (3.78)	736 (3.78)	
35-44	1,633 (8.36)	9,604 (10.01)		1,615 (8.29)	1,615 (8.29)	
45-54	5,742 (29.38)	29,016 (30.23)		5,726 (29.37)	5,726 (29.37)	
55+	11,288 (57.76)	51,018 (53.16)		11,277 (57.85)	11,277 (57.85)	
Gender, n (%)			<0.0001			1.0000
Male	10,711 (54.81)	55,177 (57.49)		10,692 (54.85)	10,692 (54.85)	
Female	8,831 (45.19)	40,793 (42.51)		8,801 (45.15)	8,801 (45.15)	
Preoperative disease characteristics						
Ascites, n (%)	4,728 (24.19)	16,808 (17.51)	<0.0001	4,691 (24.07)	4,814 (24.70)	0.1468
HE, n (%)	2,264 (11.59)	7,020 (7.31)	<0.0001	2,235 (11.47)	2,269 (11.64)	0.5901
HRS, n (%)	289 (1.48)	768 (0.80)	<0.0001	281 (1.44)	270 (1.39)	0.6370
SBP, n (%)	394 (2.02)	1,047 (1.09)	<0.0001	386 (1.98)	400 (2.05)	0.6139
Esophageal varices, n (%)	4,612 (23.60)	19,379 (20.19)	<0.0001	4,585 (23.52)	4,615 (23.68)	0.7205
Portal hypertension, n (%)	3,982 (20.38)	15,797 (16.46)	<0.0001	3,954 (20.28)	3,932 (20.17)	0.7815
Shock, n (%)				166 (0.85)	128 (0.66)	0.0261
AHRF, n (%)				1,473 (7.56)	1,238 (6.35)	<0.0001
AKI, n (%)				2,296 (11.78)	2,070 (10.62)	0.0003
Pneumonia, n (%)				2,539 (13.03)	2,303 (11.81)	0.0003
Aspiration, n (%)				259 (1.33)	226 (1.16)	0.1316
Intracerebral bleeding, n (%)				166 (0.85)	83 (0.43)	<0.0001
Intracerebral thrombosis, n (%)				81 (0.42)	40 (0.21)	0.0002
PE, n (%)				16 (0.08)	13 (0.07)	0.5773
Ventilator dependence, n (%)				124 (0.64)	118 (0.61)	0.6988
Septic shock, n (%)				282 (1.45)	231 (1.19)	0.0234
Sepsis, n (%)				842 (4.32)	704 (3.61)	0.0003
ATN, n (%)				480 (2.46)	495 (2.54)	0.6266
Smoking, n (%)	3,571 (18.27)	16,919 (17.63)	0.0317	3,562 (18.27)	3,671 (18.83)	0.1556
Alcohol use disorder, n (%)	3,167 (16.21)	15,590 (16.24)	0.8941	3,157 (16.20)	3,282 (16.84)	0.0882

Comparison of baseline variables between groups after propensity matching for specific demographics, including age, gender, region, insurance, alcohol, and smoking status, showed that the cirrhosis with surgery group had significantly higher rates of baseline comorbidities. These included the following analyzed baseline preoperative features: shock (0.85% vs. 0.66%), AHRF (7.56% vs. 6.35%), AKI (11.78% vs. 10.62), pneumonia (13.03% vs. 11.81%), intracerebral bleeding (0.85% vs. 0.43%), intracerebral thrombosis (0.42% vs. 0.21%), septic shock (1.45% vs. 1.19%), and sepsis (4.32% vs. 3.61%), all P-values significant. 

Postoperative clinical outcomes

Analyzing the six-month follow-up period for between-group differences after index (Table 3) indicated that those patients in the cirrhosis with surgery group had higher mortality compared with cirrhosis without surgery group (4.68% vs. 2.38%), a finding that persisted after propensity matching (4.67% vs. 3.51%). Comparing additional outcomes within six months after index, the cirrhosis with surgery group again had significantly increased rates of adverse hepatic outcomes, including ascites (10.60% vs. 6.07%), HE (5.00% vs. 2.50%), SBP (0.64% vs. 0.25%), HRS (0.74% vs. 0.26%), esophageal varices (5.64% vs. 4.18%), and portal hypertension (4.88% vs. 3.00%). There was a higher occurrence of septic shock, AHRF, pneumonia aspiration, intracerebral bleeding, and PE. These characteristics were similar after propensity matching, with consistently higher prevalence in the cirrhosis with surgery group for all described features in Table 3.

**Table 3:** Postoperative outcomes for matched cirrhotics with and without surgery. Variables accounted for within 30 days of surgery date for matched groups undergoing surgery compared with 30 days of initial index enrollment for cirrhotics without surgery. HE: hepatic encephalopathy; AHRF: acute hypoxemic respiratory failure; AKI: acute kidney injury; HRS: hepatorenal syndrome; PE: pulmonary embolism; ATN: acute tubular necrosis; SBP: spontaneous bacterial peritonitis.

Baseline characteristics	Cholecystectomy	Appendectomy	Gastrectomy	Colectomy	Hernia repair	Mastectomy	Aortic valve replacement	Total knee replacement	Total hip replacement
	(n=3,994)	(n=1,984)	(n=1,058)	(n=517)	(n=264)	(n=143)	(n=147)	(n=1,013)	(n=61)
Age (years), mean (SD)	51.17 (10.09)	51.43 (10.10)	55.67 (7.23)	53.99 (9.39)	53.75 (9.46)	54.54 (7.35)	54.20 (8.04)	57.58 (4.73)	55.80 (6.22)
Gender, n (%)									
Male	1,879 (47.05)	1,163 (58.62)	621 (58.70)	284 (54.93)	241 (91.29)	2 (1.40)	60 (40.82)	500 (49.36)	39 (63.93)
Female	2,115 (52.95)	821 (41.38)	437 (41.30)	233 (45.07)	23 (8.71)	141 (98.60)	87 (59.18)	513 (50.64)	22 (36.07)
Ascites, n (%)	405 (10.14)	273 (13.76)	291 (27.50)	153 (29.59)	75 (28.41)	20 (13.99)	21 (14.29)	119 (11.75)	15 (24.59)
HE, n (%)	131 (3.28)	126 (6.35)	229 (21.64)	60 (11.61)	20 (7.58)	6 (4.20)	14 (9.52)	68 (6.71)	6 (9.84)
HRS, n (%)	11 (0.28)	13 (0.66)	21 (1.98)	6 (1.16)	1 (0.38)	0	1 (0.68)	4 (0.39)	0
SBP, n (%)	19 (0.48)	14 (0.71)	28 (2.65)	20 (3.87)	5 (1.89)	0	3 (2.04)	7 (0.69)	2 (3.28)
Esophageal varices, n (%)	396 (9.91)	365 (18.40)	266 (25.14)	108 (20.89)	71 (26.89)	20 (13.99)	18 (12.24)	207 (20.43)	18 (29.51)
Portal hypertension, n (%)	377 (9.44)	289 (14.57)	235 (22.21)	115 (22.24)	48 (18.18)	23 (16.08)	21 (14.29)	170 (16.78)	16 (26.23)
Postoperative outcomes									
Death, n (%)	57 (1.43)	51 (2.57)	67 (6.33)	31 (6.00)	8 (3.03)	2 (1.40)	5 (3.40)	24 (2.37)	0
Ascites, n (%)	232 (5.81)	61 (3.07)	111 (10.49)	90 (17.41)	19 (7.20)	4 (2.80)	3 (2.04)	10 (0.99)	4 (6.56)
HE, n (%)	52 (1.30)	21 (1.06)	137 (12.95)	26 (5.03)	2 (0.76)	0	4 (2.72)	13 (1.28)	1 (1.64)
HRS, n (%)	5 (0.13)	1 (0.05)	4 (0.38)	4 (0.77)	0	0	1 (0.68)	0	0
SBP, n (%)	11 (0.28)	1 (0.05)	6 (0.57)	7 (1.35)	1 (0.38)	0	0	0	0
Esophageal varices, n (%)	78 (1.95)	40 (2.02)	65 (6.14)	24 (4.64)	10 (3.79)	4	3 (2.04)	20 (1.97)	1 (1.64)
Portal hypertension, n (%)	92 (2.30)	30 (1.51)	53 (5.01)	30 (5.80)	3 (1.14)	1 (0.70)	1 (0.68)	17 (1.68)	1 (1.64)
Shock, n (%)	2 (0.05)	2 (0.10)	4 (0.38)	8 (1.55)	1 (0.38)	0	0	0	0
AHRF, n (%)	61 (1.53)	15 (0.76)	75 (7.09)	64 (12.38)	4 (1.52)	0	1 (0.68)	15 (1.48)	0
AKI, n (%)	75 (1.88)	18 (0.91)	70 (6.62)	59 (11.41)	4 (1.52)	1 (0.70)	0	26 (2.57)	5 (8.20)
Pneumonia, n (%)	74 (1.85)	21 (1.06)	61 (5.77)	37 (7.16)	1 (0.38)	1 (0.70)	1 (0.68)	9 (0.89)	1 (1.64)
Aspiration, n (%)	8 (0.20)	0	18 (1.70)	5 (0.97)	1 (0.38)	0	0	1 (0.10)	0
Intracerebral bleeding, n (%)	0	1 (0.05)	69 (6.52)	1 (0.19)	0	0	0	3 (0.30)	0
Intracerebral thrombosis, n (%)	0	1 (0.05)	31 (2.93)	0	0	0	1 (0.68)	0	0
PE, n (%)	1 (0.03)	0	0	0	0	0	2 (1.36)	1 (0.10)	0
Ventilator dependence, n (%)	9 (0.23)	0	9 (0.85)	9 (1.74)	0	0	0	0	0
Septic shock, n (%)	11 (0.28)	4 (0.20)	7 (0.66)	14 (2.71)	1 (0.38)	1 (0.70)	0	0	0
Sepsis, n (%)	43 (1.08)	10 (0.50)	21 (1.98)	32 (6.19)	1 (0.38)	2 (1.40)	0	0	3 (4.92)
ATN, n (%)	37 (0.93)	5 (0.25)	6 (0.57)	13 (2.51)	1 (0.38)	0	1 (0.68)	8 (0.79)	1

Subgroup analysis by surgical subtype, including baseline characteristics and six-month postoperative outcomes, is listed in Table 4. Out of the initial surgical cohort of 19,493 patients, 9,181 patients were extracted from the available database by surgical subtype. The largest number of surgeries were cholecystectomies (3,994) and appendectomies (1,984), with a smaller proportion of gastrectomies (1,058), colectomies (517), total knee replacements (1,013), and hernia repairs (264). Females made up at least 36% of all the surgical subgroups except for hernia repair, including 52% of the population for cholecystectomies, 41% of appendectomies, 41% of gastrectomies, and 45% of colectomies. Patients undergoing gastrectomy, colectomy, hernia repair, and total hip replacement had the highest rates of preoperative cirrhosis complications. Among these groups, ascites was present in 28% of patients before undergoing hernia repair, 29% of those before undergoing colectomy, and 27% of those before undergoing gastrectomy, while cholecystectomy, appendectomy, and total knee replacement had relatively lower rates at baseline (10%, 13%, and 11%, respectively). Preexisting HE was found in 21% of gastrectomy patients, with lower rates for colectomy (11%), total hip replacement (9%), and lowest rate in the cholecystectomy group (3.2%).

**Table 4:** Baseline characteristics and outcomes by surgical subtypes. Postoperative outcomes were for new variable incidence within six months of surgery (i.e., only new diagnoses of ascites, HE, etc. were captured while those with preexisting complications were excluded). HE: hepatic encephalopathy; AHRF: acute hypoxemic respiratory failure; AKI: acute kidney injury; HRS: hepatorenal syndrome; PE: pulmonary embolism; ATN: acute tubular necrosis; SBP: spontaneous bacterial peritonitis.

Baseline characteristics	Cholecystectomy	Appendectomy	Gastrectomy	Colectomy	Hernia repair	Mastectomy	Aortic valve replacement	Total knee replacement	Total hip replacement
	(n=3,994)	(n=1,984)	(n=1,058)	(n=517)	(n=264)	(n=143)	(n=147)	(n=1,013)	(n=61)
Age (years), mean (SD)	51.17 (10.09)	51.43 (10.10)	55.67 (7.23)	53.99 (9.39)	53.75 (9.46)	54.54 (7.35)	54.20 (8.04)	57.58 (4.73)	55.80 (6.22)
Gender, n (%)									
Male	1,879 (47.05)	1,163 (58.62)	621 (58.70)	284 (54.93)	241 (91.29)	2 (1.40)	60 (40.82)	500 (49.36)	39 (63.93)
Female	2,115 (52.95)	821 (41.38)	437 (41.30)	233 (45.07)	23 (8.71)	141 (98.60)	87 (59.18)	513 (50.64)	22 (36.07)
Ascites, n (%)	405 (10.14)	273 (13.76)	291 (27.50)	153 (29.59)	75 (28.41)	20 (13.99)	21 (14.29)	119 (11.75)	15 (24.59)
HE, n (%)	131 (3.28)	126 (6.35)	229 (21.64)	60 (11.61)	20 (7.58)	6 (4.20)	14 (9.52)	68 (6.71)	6 (9.84)
HRS, n (%)	11 (0.28)	13 (0.66)	21 (1.98)	6 (1.16)	1 (0.38)	0	1 (0.68)	4 (0.39)	0
SBP, n (%)	19 (0.48)	14 (0.71)	28 (2.65)	20 (3.87)	5 (1.89)	0	3 (2.04)	7 (0.69)	2 (3.28)
Esophageal varices, n (%)	396 (9.91)	365 (18.40)	266 (25.14)	108 (20.89)	71 (26.89)	20 (13.99)	18 (12.24)	207 (20.43)	18 (29.51)
Portal hypertension, n (%)	377 (9.44)	289 (14.57)	235 (22.21)	115 (22.24)	48 (18.18)	23 (16.08)	21 (14.29)	170 (16.78)	16 (26.23)
Postoperative outcomes									
Death, n (%)	57 (1.43)	51 (2.57)	67 (6.33)	31 (6.00)	8 (3.03)	2 (1.40)	5 (3.40)	24 (2.37)	0
Ascites, n (%)	232 (5.81)	61 (3.07)	111 (10.49)	90 (17.41)	19 (7.20)	4 (2.80)	3 (2.04)	10 (0.99)	4 (6.56)
HE, n (%)	52 (1.30)	21 (1.06)	137 (12.95)	26 (5.03)	2 (0.76)	0	4 (2.72)	13 (1.28)	1 (1.64)
HRS, n (%)	5 (0.13)	1 (0.05)	4 (0.38)	4 (0.77)	0	0	1 (0.68)	0	0
SBP, n (%)	11 (0.28)	1 (0.05)	6 (0.57)	7 (1.35)	1 (0.38)	0	0	0	0
Esophageal varices, n (%)	78 (1.95)	40 (2.02)	65 (6.14)	24 (4.64)	10 (3.79)	4	3 (2.04)	20 (1.97)	1 (1.64)
Portal hypertension, n (%)	92 (2.30)	30 (1.51)	53 (5.01)	30 (5.80)	3 (1.14)	1 (0.70)	1 (0.68)	17 (1.68)	1 (1.64)
Shock, n (%)	2 (0.05)	2 (0.10)	4 (0.38)	8 (1.55)	1 (0.38)	0	0	0	0
AHRF, n (%)	61 (1.53)	15 (0.76)	75 (7.09)	64 (12.38)	4 (1.52)	0	1 (0.68)	15 (1.48)	0
AKI, n (%)	75 (1.88)	18 (0.91)	70 (6.62)	59 (11.41)	4 (1.52)	1 (0.70)	0	26 (2.57)	5 (8.20)
Pneumonia, n (%)	74 (1.85)	21 (1.06)	61 (5.77)	37 (7.16)	1 (0.38)	1 (0.70)	1 (0.68)	9 (0.89)	1 (1.64)
Aspiration, n (%)	8 (0.20)	0	18 (1.70)	5 (0.97)	1 (0.38)	0	0	1 (0.10)	0
Intracerebral bleeding, n (%)	0	1 (0.05)	69 (6.52)	1 (0.19)	0	0	0	3 (0.30)	0
Intracerebral thrombosis, n (%)	0	1 (0.05)	31 (2.93)	0	0	0	1 (0.68)	0	0
PE, n (%)	1 (0.03)	0	0	0	0	0	2 (1.36)	1 (0.10)	0
Ventilator dependence, n (%)	9 (0.23)	0	9 (0.85)	9 (1.74)	0	0	0	0	0
Septic shock, n (%)	11 (0.28)	4 (0.20)	7 (0.66)	14 (2.71)	1 (0.38)	1 (0.70)	0	0	0
Sepsis, n (%)	43 (1.08)	10 (0.50)	21 (1.98)	32 (6.19)	1 (0.38)	2 (1.40)	0	0	3 (4.92)
ATN, n (%)	37 (0.93)	5 (0.25)	6 (0.57)	13 (2.51)	1 (0.38)	0	1 (0.68)	8 (0.79)	1

Regarding six-month postsurgical outcomes by surgical subtype, death was highest in the gastrectomy (6.33%) and colectomy (6.0%) groups, with lower rates for surgical aortic valve replacement (3.40%), hernia repair (3.03%), appendectomy (2.57%), total knee replacement (2.37%), cholecystectomy (1.43%), and total hip replacement (0 deaths out of 61 patients). Rates of new hepatic decompensating events within 30 days were comparatively higher for gastrectomy and colectomy, including for ascites after colectomy (17.4%), gastrectomy (10.4%), new HE (5.03%, 12.95%), HRS (0.77%, 0.38%), SBP (1.35%, 0.57%), and esophageal varices (4.64%, 6.14%). Among the other outcomes measured, high rates of respiratory failure, AKI, and sepsis were noted in the colectomy and gastrectomy groups. Intracerebral bleeding was particularly high by comparison in the gastrectomy group (6.52%), with all other groups having <0.30%. Comparison to control cirrhotics without surgery was not available for the groups by surgical subtype, so significant differences with controls (including P-values) could not be reported.

Healthcare utilization and costs

Healthcare utilization was compared between the six-month baseline period (presurgery) and the six months following the index date (postsurgery) in the 19,493 cirrhotic patients who underwent surgery (Table 5). The total number of claims was significantly higher in the six-month period after surgery compared to the baseline six months prior to surgery (before vs. after, 29.51 vs. 38.11), representing an increase of 8.60 claims after surgery. The six-month period following surgery was characterized by a significantly higher number of inpatient admissions (6.05 vs. 3.18), outpatient visits (19.72 vs. 15.60), and pharmaceutical claims (11.76 vs. 10.12) compared to the six-month period before surgery. Translating these claims into costs, the total cost of healthcare services in a 6-month period was significantly higher before surgery than after surgery (pre vs. post, $38,116 vs. $58,246), representing a post-diagnosis increase in healthcare expenditures of $20,130 (Table 5). These higher costs are predominantly due to inpatient admissions ($21,667 vs. 34,446), followed by higher costs for outpatient visits ($11,871 vs. 18,104) and pharmaceutical cost (3,501 vs. 4,643), with all P-values significant.

**Table 5:** Healthcare resource utilization and healthcare costs before and after of surgery in patients with cirrhosis. ED: emergency department; LoS: length of stay. *All values presented include (mean, standard deviation) unless otherwise specified.

Healthcare utilization	Presurgery		Postsurgery	Difference (95% CI)	P-value*
	(n=19,493)		(n=19,493)		
Total claims per patient	29.51 (23.27)		38.11 (30.30)	8.60 (8.26, 8.94)	<0.0001
Inpatient admissions					
Prevalence of at least one visit, n (%)	7,107 (36.46)		10,064 (51.63)	0.15 (0.14, 0.16)	<0.0001
Number of admissions	3.18 (8.57)		6.05 (13.95)	2.86 (2.67, 3.05)	<0.0001
Total LoS	4.32 (12.17)		4.99 (14.14)	0.66 (0.44, 0.89)	<0.0001
ED visits					
Prevalence of at least one visit, n (%)	6,492 (33.30)		5,716 (29.32)	-0.04 (-0.05, -0.03)	<0.0001
Number of visits	0.62 (1.42)		0.59 (1.49)	-0.03 (-0.05, 0.00)	<0.0001
Outpatient visits					
Prevalence of at least one visit, n (%)	19,309 (99.06)		19,416 (99.60)	0.01 (0.00, 0.01)	<0.0001
Number of visits	15.60 (13.72)		19.72 (17.15)	4.12 (3.92, 4.33)	<0.0001
Pharmaceutical claims					
Number of claims	10.12 (10.11)		11.76 (10.90)	1.64 (1.55, 1.73)	<0.0001
Healthcare costs					
Total cost	38,116 (82,010)		58,246 (124,467)	20,130 (18370, 21890)	<0.0001
Inpatient cost	21,667 (71,788)		34,446 (109,954)	12,778 (11141, 14415)	<0.0001
ED cost	1,077 (4,418)		1,053 (3,912)	-24 (-94, 47)	<0.0001
Outpatient cost	11,871 (25,029)		18,104 (35,914)	6,233 (5786, 6679)	<0.0001
Pharmaceutical cost	3,501 (12,590)		4,643 (15,823)	1,143 (915, 1370)	<0.0001

Healthcare utilization was compared next between patients with a record of cirrhosis with surgery and cirrhosis without surgery (Table 6). The total number of claims in the six months after surgery was higher per patient in the cirrhosis with surgery group than in the cirrhosis without surgery (38.11 vs. 28.64), representing an increase of 9.48 claims per patient within the six months after index period. The post six-month period was characterized by significantly higher numbers of inpatient admissions (6.05 vs. 2.35), outpatient visits (19.72 vs. 15.23), and pharmaceutical claims (11.76 vs. 10.61) in the cirrhosis with surgery group compared to cirrhosis without surgery, respectively. The prevalence of at least one inpatient admission was significantly higher in the cirrhosis with surgery group compared to the cirrhosis without surgery during this period (51.63% vs. 22.32%). The average length of inpatient stay in the cirrhosis with surgery cohort was higher (4.99 days vs. 2.09 days) compared to the cirrhosis without surgery cohort, with all P-values significant. Healthcare costs were compared and analyzed between matched cirrhotics with surgery cases and cirrhosis-only controls (Table 6). In the analysis, the per-patient total costs of healthcare services within six months after index were US $58,246 and $30,400 for cirrhosis with surgery and cirrhosis without surgery, respectively (Table 6). The difference in the total unadjusted cost was significant, with cirrhosis with surgery cases costing $27,846 more than cirrhosis without surgery cases. The average difference between groups in healthcare costs within the six-month post-index period was $22,000 from inpatient costs, $337 from ED costs, and $5,879 from outpatient visits (all within narrow confidence intervals).

**Table 6:** Healthcare resource utilization and cost differences between cirrhotics undergoing surgery compared with cirrhotics after index diagnosis without surgery in six month follow-up period. ED: emergency department; LoS: length of stay. Values reported with (mean, standard deviation) unless stated otherwise.

Healthcare utilization	Cirrhosis with surgery	Cirrhosis without surgery	Difference (95% CI)	P-value
	(n=19,493)	(n=19,493)		
Total number of claims	38.11 (30.30)	28.64 (25.26)	9.48 (8.97, 9.98)	<0.0001
Inpatient admissions				
Prevalence of at least one visit, n (%)	10,064 (51.63)	4,351 (22.32)	0.29 (0.28, 0.30)	<0.0001
Number of admissions	6.05 (13.95)	2.35 (8.16)	3.69 (3.48, 3.91)	<0.0001
Total LoS (days)	4.99 (14.14)	2.09 (8.38)	2.89 (2.67, 3.12)	<0.0001
ED visits				
Prevalence of at least one visit, n (%)	5,716 (29.32)	4,442 (22.79)	0.07 (0.06, 0.07)	<0.0001
Number of visits	0.59 (1.49)	0.45 (1.33)	0.14 (0.12, 0.17)	<0.0001
Outpatient visits				
Prevalence of at least one visit, n (%)	19,416 (99.60)	19,228 (98.64)	0.01 (0.01, 0.01)	<0.0001
Number of visits	19.72 (17.15)	15.23 (16.16)	4.49 (4.19, 4.80)	<0.0001
Pharmaceutical claims				
Number of claims	11.76 (10.90)	10.61 (10.46)	1.14 (0.94, 1.35)	<0.0001
Healthcare cost ($)				
	Total cost	58,246 (124,467)	30,400 (83,533)	27,846 (25829, 29863)	<0.0001
Inpatient cost	34,446 (109,954)	12,391 (68,561)	22,055 (20283, 23827)	<0.0001
ED cost	1,053 (3,912)	716 (2,828)	337 (270, 405)	<0.0001
Outpatient cost	18,104 (35,914)	12,225 (32,015)	5,879 (5231, 6526)	<0.0001
Pharmaceutical cost	4,643 (15,823)	5,068 (16,763)	-425 (-746, -103)	0.0844

Finally, healthcare utilization and cost analyses were performed on the nine different surgical subtypes extracted (Table 7). Total claims per patient were highest for total hip replacement (57.59), followed by total knee replacement (50.80), gastrectomy (50.69), colectomy (50.16), and mastectomy (41.93). Cholecystectomy, the most common surgical type, had 25.36 total claims per patient. A high percentage of the orthopedic procedure patients had at least one inpatient admission (96.7% of total hip replacement and 97.5% of total knee replacement patients), with average length of stay (LoS) of 9.97 days and 5.01 days, respectively. Colectomy also had very high rates of inpatient stay (89.56%), with long average LoS (10.57 days). High number of admissions per patient were seen for colectomy (12.20), gastrectomy (11.29), and total hip replacement (10.48). Lower number of admissions and shorter rates of stay were seen for cholecystectomy (37.8%, LoS 2.11 days), appendectomy (24.04%, LoS 1.43 days), and hernia repair (20.8%, LoS 1.43 days). Total costs for these surgeries (Table 7) were highest per patient in total hip replacement ($99,807), colectomy ($98,057), mastectomy ($73,759), gastrectomy ($68,523), and gastrectomy ($68,523). A large percentage of these costs was again driven by inpatient care, including $75,768 (out of $99,807) for THR, $66,756 (of $98,057) for colectomy, and $42,772 (of $68,523) for gastrectomy. Procedures that are often done outpatient, including cholecystectomy, had a lower proportion of inpatient costs ($14,361 of $33,483) with similar outpatient costs. Mastectomy had a notably higher total and proportion of outpatient cost ($45,791 out of $73,759), accounting for some of its relatively high total costs. ED costs and pharmaceutical costs were similar between surgeries, although they still contributed significantly to health claims and costs (Table 5).

**Table 7:** Healthcare utilization and cost in the six-month follow-up period among cirrhotics by surgical subtype. ED: emergency department; LoS: length of stay. Values reported as (mean, standard deviation) unless otherwise specified.

Healthcare utilization	Cholecystectomy	Appendectomy	Gastrectomy	Colectomy	Hernia repair	Mastectomy	Aortic valve replacement	Total knee replacement	Total hip replacement
	(n=3,994)	(n=1,984)	(n=1,058)	(n=517)	(n=264)	(n=143)	(n=147)	(n=1,013)	(n=61)
Total number of claims	25.36 (22.19)	28.04 (21.53)	50.69 (36.52)	50.16 (35.33)	23.75 (19.91)	41.93 (21.01)	34.77 (25.29)	50.80 (23.26)	57.59 (34.91)
Inpatient admissions									
Prevalence of at least one visit, n (%)	1,513 (37.88)	477 (24.04)	696 (65.78)	463 (89.56)	55 (20.83)	92 (64.34)	33 (22.45)	988 (97.53)	59 (96.72)
Number of admissions	2.81 (8.98)	1.60 (5.48)	11.29 (20.71)	12.20 (18.14)	1.60 (4.44)	2.26 (5.01)	2.31 (6.28)	4.98 (7.83)	10.48 (18.25)
Total LoS	2.11 (8.13)	1.43 (5.80)	9.86 (20.59)	10.47 (16.65)	1.43 (4.16)	1.97 (4.96)	1.93 (5.63)	5.01 (8.09)	9.97 (17.36)
ED visits									
Prevalence of at least one visit, n (%)	957 (23.96)	484 (24.40)	464 (43.86)	171 (33.08)	61 (23.11)	45 (31.47)	53 (36.05)	242 (23.89)	24 (39.34)
Number of visits	0.45 (1.22)	0.45 (1.29)	1.02 (2.10)	0.70 (1.39)	0.41 (1.02)	0.60 (1.13)	0.60 (1.09)	0.41 (1.01)	0.85 (1.47)
Outpatient visits									
Prevalence of at least one visit, n (%)	3,970 (99.40)	1,978 (99.70)	1,056 (99.81)	511 (98.84)	264 (100.00)	143 (100.00)	147 (100.00)	1,013 (100.00)	61 (100.00)
Number of visits	12.28 (11.76)	15.39 (12.89)	25.40 (20.23)	25.53 (22.28)	12.00 (12.60)	24.89 (14.16)	18.48 (15.41)	30.55 (15.47)	31.59 (18.88)
Pharmaceutical claims	9.82 (9.50)	10.60 (10.32)	12.97 (11.59)	11.74 (10.16)	9.74 (9.31)	14.18 (10.75)	13.38 (11.68)	14.86 (11.63)	14.67 (11.08)
Healthcare cost ($)									
	Total cost	33,483 (66,094)	25,059 (50,110)	68,523 (109,796)	98,057 (131,983)	34,325 (50,107)	73,759 (58,065)	30,493 (46,867)	63,235 (98,873)	99,807 (89,742)
Inpatient cost	14,361 (57,124)	8,961 (38,215)	42,772 (96,234)	66,746 (113,775)	8,365 (24,593)	22,322 (28,052)	11,158 (32,506)	43,872 (58,722)	75,768 (76,433)
ED cost	927 (3,270)	1,167 (5,832)	1,664 (4,347)	1,169 (2,979)	753 (1,895)	1,413 (6,451)	1,115 (2,650)	646 (2,809)	1,268 (2,696)
Outpatient cost	14,973 (20,333)	10,475 (16,427)	19,174 (37,163)	26,302 (53,791)	20,874 (36,569)	45,791 (49,568)	12,689 (22,744)	14,148 (49,750)	17,342 (32,937)
Pharmaceutical cost	3,222 (11,922)	4,455 (14,598)	4,912 (16,174)	3,840 (9,489)	4,333 (13,913)	4,234 (13,604)	5,531 (17,088)	4,569 (29,070)	5,429 (11,144)

## Discussion

This retrospective analysis of insured United States adults aimed to identify and compare baseline risk features in cirrhotics who did and did not undergo surgery, compare basic outcomes across groups in a six-month follow-up period, and perform a healthcare burden and cost analysis of these groups. We also performed subgroup analysis by surgical subtypes for nine major categories of surgeries and again performed outcomes, utilization, and cost analysis to help differentiate outcomes and costs by different types of surgery. A baseline cirrhotic group that did not undergo surgery was included to help compare historical features and basic outcomes in a similar time frame. Our analysis included 19,493 cirrhotic patients who underwent surgery, a very large cohort in comparison to previous series, which adds depth to this study in identifying perioperative and outcomes not previously noted (for reference, the Mayo score derivation included 772 patients and the VOCAL-Penn cohort 4,712 in their multivariate analyses). Our records-based analysis allowed for the identification of outcomes not identified in previous retrospective series. This included several non-hepatic outcomes of significant clinical importance, including intracerebral hemorrhage and septic shock. The population demographic was also more balanced than previous series, as it included a large subset of insured United States adults based on claims data. In the groups listed, females represented 45%-57% of the population in the compared cirrhosis groups, a much more balanced proportion than in previous series.

With regard to baseline features, the cirrhotic patients who underwent surgery had significantly more baseline comorbidities when compared with cirrhotic patients who did not undergo surgery. This included baseline complications of liver disease, including ascites, hepatic encephalopathy, SBP, and HRS, as well as all other baseline features analyzed, including history of septic shock, pneumonia, venous thromboembolism, and acute hypoxic respiratory failure. The CCI analysis showed that a higher proportion of patients had at least one comorbidity in the cirrhotic with surgery group compared with group without surgery (68.06% and 52.30%). This higher burden of sick patients in the cirrhosis group is likely explained in part by the process of indexing cirrhotic patients in the non-surgery group based on first diagnosis, where these patients are likely to have less advanced disease compared with those patients who are undergoing evaluation before selection for surgery where cirrhosis was first discovered. It is also possible that a proportion of these surgeries were undertaken for urgent or emergent surgical issues, accounting for the sicker baseline population. Considering there were more total surgeries in the surgical cohort, it may be the case that a single surgery precipitated more decompensations and the need for additional interventions. Overall, this indicates that there is a significant portion of patients with more advanced liver disease undergoing a broad range of surgeries in this cohort, as the presence of ascites (25.15% vs. 17.51%) and hepatic encephalopathy (11.42% vs. 7.31%) implies many patients with more advanced portal hypertension and more advanced Child-Pugh scoring. Lab values were not available from this type of database analysis, which prevented the calculation of MELD or CPC from this population.

 In the analyzed six-month post-index period for matched patients (Table 3), significantly more patients in the cirrhosis with surgery group experienced adverse clinical outcomes compared with controls without cirrhosis, including a higher percentage of death (4.68% vs. 2.38%). This mortality rate was lower than seen in previous series (often cited 5%-50% perioperative mortality based on MELD [8]), which may reflect improved perioperative management in this newer series compared with older ones, or that by looking at the population at large there is less bias towards those cirrhotic patients prone to poor outcomes, indicating overestimation of mortality in older smaller series. More patients in the surgical arm developed new ascites (10.6% vs. 6.07%), new hepatic encephalopathy (5.00% vs. 2.50%), HRS (0.75 vs. 0.26%), and SBP (0.65% vs. 0.25), with all P values <0.0001. It should be noted that this analysis includes new diagnoses of hepatic decompensating events in the post-index period and excludes patients with a diagnosis of previous decompensating events. A significantly higher proportion of patients in the surgical cohort already had these features at baseline. Patients in the cirrhosis with surgery group experienced a higher prevalence of shock, sepsis, acute hypoxemic respiratory failure, AKI, pneumonia, intracerebral bleeding/thrombosis, and PE within six months after the index date compared to the cirrhotic patients who did not undergo surgery. When analyzing outcomes by surgical subtype (Table 4), the worst clinical outcomes were consistently seen for colectomy and gastrectomy, including highest mortality rates (6.00% and 6.33%) for both of these groups, with higher rates of new hepatic decompensating features including ascites, hepatic encephalopathy, HRS, SBP, and esophageal varices. Mortality rates for cholecystectomy and appendectomy were relatively lower, at 1.43% and 2.57%, respectively. Gastrectomy had a very high percentage of intracerebral bleeding (6.52%), with no other surgery having more than 0.30%. A higher proportion of patients in the gastrectomy and colectomy groups had preexisting decompensating hepatic features, indicating that a significant percentage of these patients were likely having surgery at a more advanced stage or for urgent or emergent complications such as bowel perforation. These open intra-abdominal surgeries had the highest rates of new complications in the postoperative period, especially compared with the less invasive and often elective surgical subtypes including cholecystectomy, hernia repair, aortic valve replacement, and total knee replacement. Coding by type of surgical approach (open vs. laparoscopic) for the various intra-abdominal surgery was not performed, which does limit generalizability for this subgroup. Relatively high comorbidities (including hepatic disease) at baseline were seen for total hip replacement, but postoperative outcomes were less severe than intra-abdominal surgeries. The statistical significance of these outcomes compared with the control cirrhotic group without surgery was not able to be performed using the available dataset, which limits the analysis to relative rates by outcome for surgical subtype.

The health utilization and cost analysis illustrate that surgery in patients with cirrhosis is associated with significantly higher healthcare use and cost burden in the six-month postoperative period. In the six months before and after surgery, the burden of a new surgery diagnosis was demonstrated by an increase in the total number of claims by 8.6 and an increase in total annual healthcare costs of $20,130. Compared to cirrhotics without surgery, the surgical group had significantly higher costs in the six-month follow-up period. Over half (51.63%) of cirrhotic patients who underwent surgery had at least one inpatient admission in the six-month follow-up period compared to 36.46% of cirrhotic patients without surgery. Patients with cirrhosis who underwent surgery had a significantly higher number of admissions (2.86) compared to cirrhotics who did not undergo surgery. The increase in healthcare utilization and claims was primarily attributed to inpatient admissions (2.86), outpatient visits (4.12), and pharmaceutical claims (1.64). An increase in healthcare costs was primarily attributed to and evident by an increase in inpatient cost ($12,778), outpatient cost ($6,233), and pharmaceutical cost ($1,143). These findings are unsurprising, as patients require inpatient admission during or after their surgery, close outpatient follow-up, and may require re-admission due to further intervention, complications, or otherwise unrelated decompensation of their comorbidities, as they represent a very fragile population. It is also likely that additional surgeries and procedures were required for patients whose surgeries prompted decompensation, reflected by the total increase in the proportion of patients requiring surgery in the surgical cohort.

Regarding healthcare utilization, the case-control analysis showed a significant increase in healthcare use (9.48 claims per patient) and costs ($27,846 per patient) in cirrhotic patients who had undergone surgery compared to cirrhotic patients who had not. The differences in utilization and costs were primarily driven by increased inpatient admission claims (3.691) and costs ($22,055) and outpatient visits (4.49) and costs ($5,879). Patients with cirrhosis who underwent surgery showed a significantly higher prevalence of at least one inpatient admission (51.63% vs. 22.32%) and ED visit (29.32% vs. 22.79%). Patients with cirrhosis who underwent surgery had a significantly higher number of inpatient admissions (3.69) and total length of stay (2.89) compared to cirrhotics who did not undergo surgery. These findings are unsurprising, and these data help put into easily understood claim and dollar figures the magnitude of increased costs that are associated with surgery in cirrhosis, especially the proportion of costs driven by inpatient care.

Regarding utilization and cost in the subgroup analysis by surgical type, claims and costs were highest in patients undergoing total hip replacement, colectomy, gastrectomy, and total knee replacement (Table 7). Again, the costs of these procedures were driven by a high proportion of inpatient costs. The high morbidity and advanced disease noted for the gastrectomy, colectomy, and total hip replacement groups preoperatively help account for the increased health utilization and costs for these conditions, as they are complex morbid surgeries often associated with long inpatient stays and are often performed in patients for urgent issues such as bowel perforation or hip fracture. Total knee replacement had a relatively high cost ($63,235) in comparison with its comorbidity profile and outcomes, including high inpatient costs, which may be accounted by recovery and rehab associated with knee replacements. Mastectomy stood out with a high proportion of outpatient costs ($45,791 of $73,759), where it is likely that costs associated with breast cancer care are driving more of the health utilization than the surgery, which had relatively good clinical outcomes. Again, comparison of utilization and costs by subgroup with controls was not able to be performed from the available dataset, which does limit the strength of the subgroup analysis.

This analysis helps verify the large number of cirrhotic patients who are undergoing a variety of surgeries and shows that on average they have a significant number of both liver-related and non-liver-related comorbidities going into these procedures than cirrhotic patients who have not undergone surgery. The mortality rate was lower in the surgical group than in previous series, possibly from large sample size including some healthier patients, or advances in care in this newer series. Some of the risk factors are not accounted for in newer scoring systems and are present in significant proportions, including ascites, HE, SBP, pneumonia, sepsis, AHRF, and ATN. This analysis does not allow correlation with outcomes by risk factor but does identify these areas of interest for future series, as they were also significantly higher at baseline and in the postoperative period in the surgical group. These outcomes and comorbidities contribute to the significantly increased health utilization and healthcare costs noticed in this analysis, a substantial portion of which is driven up by the high cost of additional inpatient stays. Subgroup analysis highlights the high morbidity and mortality associated with colectomy, gastrectomy, and total hip replacement and the associated high costs of these surgeries accounted for by long inpatient hospitalizations.

There are limitations to this analysis. The cohort selected including nonalcoholic cirrhosis patients, which removes a significant proportion of cirrhotic patients with alcoholic liver disease (approximately 21% of all patients with cirrhosis) [12-14]. It also does not differentiate between elective and nonelective procedures, which weakens the associations of some of the outcomes data, as presumably many of the worst outcomes were for urgent or emergent surgeries in patients with more advanced disease. It does however strengthen the cost analysis and health burden data, as it demonstrates that many sick cirrhotic patients are still undergoing surgeries for any indication, and likely contributes significantly to the cost burden from this patient population. The analysis does not specifically follow individual patients and correlate preexisting risk factors with outcomes data, making extrapolation about the impact of these features limited, but something that could be elaborated on further in future retrospective analyses. As stated previously, comparison with the surgical subtypes with the control cirrhotic patients without surgery was not able to be performed on the dataset, which limits the strength of the outcomes and costs analyses by type of surgery.

## Conclusions

Few other studies have examined the increase in healthcare utilization and costs as well as the associated cost burden due to surgery in patients with cirrhosis. The findings in our study indicate that cirrhotic patients who have had a diagnosis or surgery or who have undergone a surgical procedure are associated with higher comorbidities, healthcare costs, and cost burden in the United States, as determined from claims data from 2007 to 2017. Future analysis should investigate carefully among these subsets of patients to help correlate if some of the adverse outcome data are due in part to those with advanced disease and the indication for surgery and to help clarify risk factor associations with outcomes as suggested by the findings in this study. Considering the growing prevalence of cirrhotic patients and sizable healthcare costs shown to be associated with these surgeries in this analysis, further study into these features will be critical.
